# COVID-19 lockdown has altered the dynamics between affective symptoms and social isolation among older adults: results from a longitudinal network analysis

**DOI:** 10.1038/s41598-021-94301-6

**Published:** 2021-07-19

**Authors:** Junhong Yu, Rathi Mahendran

**Affiliations:** 1 Department of Psychological Medicine, Mind Science Centre, Yong Loo Lin School of Medicine, National University of Singapore, NUHS Tower Block, Level 9, 1E Kent Ridge Road, Singapore, 119228 Singapore; 2 Academic Development Department, Duke-NUS Medical School, 8 College Road, Singapore, Singapore; 3 Psychology, School of Social Sciences, Nanyang Technological University, Singapore, Singapore

**Keywords:** Psychology, Human behaviour, Anxiety, Depression

## Abstract

The COVID-19 lockdown has drastically limited social interactions and brought about a climate of fear and uncertainty. These circumstances not only increased affective symptoms and social isolation among community dwelling older adults but also alter the dynamics between them. Using network analyses, we study the changes in these dynamics before and during the lockdown. Community-dwelling older adults (N = 419) completed questionnaires assessing depression, anxiety, and social isolation, before the COVID-19 pandemic, as part of a cohort study, and during the lockdown period. The total scores of these questionnaires were compared across time. For the network analyses, partial correlation networks were constructed using items in the questionnaires as nodes, separately at both timepoints. Changes in edges, as well as nodal and bridge centrality were examined across time. Depression and anxiety symptoms, and social isolation had significantly increased during the lockdown. Significant changes were observed across time on several edges. Greater connectivity between the affective and social isolation nodes at lockdown was observed. Depression symptoms have become more tightly coupled across individuals, and so were the anxiety symptoms. Depression symptoms have also become slightly decoupled from those of anxiety. These changing network dynamics reflect the greater influence of social isolation on affective symptoms across individuals and an increased vulnerability to affective disorders. These findings provide novel perspectives and translational implications on the changing mental health context amidst a COVID-19 pandemic situation.

## Introduction

The COVID-19 outbreak has brought about a global healthcare crisis on an unprecedented scale. Healthcare systems are on the brink of exhaustion as they cope with the ever-increasing number of infections. In an attempt to slow down the spread of the virus to relieve the pressure on healthcare resources, lockdown measures have been widely implemented.

Within the context in Singapore, the COVID-19 ‘lockdown’ consisted of two phases which were known locally as the ‘circuit-breaker’ and ‘phase 1’. The circuit-breaker measures were implemented on 8th April 2020. These measures included the closure of non-essential workplaces, schools, and places of worship. Eating out was no longer allowed. Food had to be taken away from food establishments and consumed at home. Residents were strongly encouraged to stay at home, unless they had to travel for essential work or needed to purchase essentials. Home-based gatherings consisting of people not within the same household were forbidden. Subsequently, some of these measures were relaxed in the phase 1 period, which spanned from 2nd to 19th June 2020. During this period, some non-essential businesses and schools could reopen, and each household could receive up to two visitors a day.

Most of these lockdown measures were not unique to Singapore. Around the world, people are strongly advised, or in some cases legally obligated to stay at home. Inevitably, these measures increased social isolation within the community. While many can, to some extent, mitigate such isolation by shifting their day to day social interactions and activities online, a sizable proportion of older adults may not be able to make this shift due to their relatively low levels of ‘digital literacy’^1^. Thus, the older adult population was arguably more vulnerable to the effects of social isolation during the lockdown. Such isolation will result in major mental health consequences. As longitudinal research has shown, perceived and objective social isolation among older adults are significant risk factors for developing depression and anxiety symptoms^2^. Nevertheless, despite their increased social isolation related concerns, research have generally indicated that older adults fared significantly better than young adults in in terms of experiencing less COVID-19-related anxiety, and less negative affect and mood states^3–5^. This may be due to the fact that older adults reported higher perceived coping efficacy and were generally more confident of the COVID-19 situation^5^.

Along with social isolation, the pandemic has also brought about a climate of fear^6^. These fears generally relate to becoming infected with the virus^7^, unknowingly infecting others^8^, losing livelihoods or stigmatization^9^. Taken together with the general uncertainty of the pandemic situation^10^, these circumstances further provoked affective symptoms. Due to these aggravating factors, we would not just expect a general increase in the levels of affective symptoms and perceived isolation, but also a change in the dynamics between them—reflecting the greater influence of social isolation and increased vulnerability to affective disorders during this period.

In the current study, we used network analyses to study how these dynamics have altered during the lockdown. A network is characterized by ‘nodes’ that are linked to each other via ‘edges’. According to the network model of psychopathology, psychiatric symptoms do not arise from psychopathology, but rather it is the dynamic interaction (edges) between psychiatric symptoms (nodes) that constitutes psychopathology^11^. Compared to conventional analyses involving total scale scores, the network approach focuses on symptom level associations, not just within disorder but also across disorders. The focus at the symptom level would provide more useful insights given the heterogeneous symptomatic presentations in psychiatric disorders such as depression^12^ and the fact that different symptoms within the same disorder are associated with different risk factors, precipitating stressors, and consequences on daily functioning^13^.

Thus far, two studies have attempted to model the interactions between affective symptoms across various time points in the COVID-19 pandemic. The first^14^ compared networks consisting of nodes representing the total scores of scales assessing depression, anxiety, stress, fear of COVID, intolerance of uncertainty, emotion regulation and social support between lockdown and post-lockdown. Among their main findings, they note that depression, anxiety, stress, fear of COVID tend to cluster together. The second^15^ compared networks consisting of nodes representing items from scales assessing depression, anxiety, trauma and COVID-19-related anxiety between two time points separated by a month, within a lockdown. In this study, it was reported that although anxiety and depression symptoms formed separate clusters in the initial time point, both types of symptoms eventually coalesced into a single contiguous cluster in the subsequent time point. Our study intends to extend the work of these two studies in two important ways. First, on top of analyzing data from the lockdown time point, we were also able to obtain data from the pre-COVID-19 time point, thus we are in a unique position to observe the changes to the network of affective symptoms as a result of the lockdown. Second, both studies were carried out in the general adult population, whereas we decided to focus on the older adult population, given their increased vulnerability to social isolation, as discussed earlier. This is particularly important given that prospective research on social isolation/loneliness among older adults during the COVID-19 pandemic have been lacking^16^.

To these ends, we constructed network consisting of nodes representing the affective and social isolation-related features before and during the lockdown, to study the changes in the network structure across time in an older adult sample. We tested the hypothesis that social isolation had a greater influence on affective symptoms at lockdown by examining the connectivity between the social isolation and affective nodes before and during the lockdown. We also tested the hypothesis that the lockdown had resulted in a general increase in connectivity among affective nodes, reflecting the increased coupling among affective symptoms and vulnerability to affective disorders.

## Methods

### Participants and procedures

Data collection was carried out across two time points—pre-COVID-19 and lockdown. In the former, participants (N = 762) were recruited from the community for an observational cohort study. The inclusion criteria and recruitment procedures for this time point have been described in detail elsewhere^17^. Data collection for this study took place from 1st February 2018 to 15th January 2020; all responses for the relevant variables were collected via pen-and-paper questionnaires. Subsequently, a follow-up study was initiated for the lockdown time point. All participants from the previous time point, except those diagnosed with dementia, were contacted (N = 614). Data collection for this follow-up study spanned from 11th May 2020 to 19th June 2020. Depending on participants’ preference, the English or back-translated Chinese questionnaires were administered via an online platform (i.e., Qualtrics) or pen-and-paper questionnaires mailed to them. They were given S$10 upon completion of the questionnaires. Ethical approval for both studies was granted by the National University of Singapore Institutional Review Board. Informed consent was obtained from participants prior to their participation in both studies. The study procedures have been performed in accordance with the Declaration of Helsinki.

A total of 419 participants (275 females) completed both waves of data collection and were included in the current analyses. They had a mean age of 69.0 years (SD = 5.5), an average of 13.6 years of education (SD = 3.8), and a mean Mini-Mental State Examination score of 28.4 (SD = 1.5). The average follow-up period was 1.3 years (SD = 0.52; range = 0.3 to 2.3). Most participants chose to complete the English questionnaires (N = 315); 231 and 190 participants completed the questionnaires via the online and offline options, respectively.

### Measures

The 15-item version^18^ of the Geriatric Depression Scale (GDS) was used to index the level of depressive symptoms. This scale consists of 15 yes/no questions, each worth a point, giving a maximum total score of 15. It has a cutoff score of 4/5. 21 and 52 subjects exceeded this cutoff score at the pre-COVID-19 and lockdown time points, respectively. This version of the GDS has demonstrated good psychometric validity in the local context^19^. It has a Cronbach alpha of 0.75 and 0.73 at the pre-COVID-19 and lockdown timepoints, respectively.

The Geriatric Anxiety Inventory (GAI)^20^ was used to measure the level of anxiety symptoms. There are 20 agree/disagree items in the questionnaire, each worth a point, giving a maximum possible total score of 20. The GAI was validated and had shown good psychometric properties in a similar Asian population^21^. It has a Cronbach alpha of 0.89 and 0.92 at the pre-COVID-19 and lockdown timepoints, respectively. The GAI has a cutoff score of 10/11. Six and 18 subjects exceeded this cutoff score at the pre-COVID-19 and lockdown time points, respectively.

We used the friendship scale (TFS) to measure social isolation. This scale was designed specifically for use among older adults and consisted of six 5-point scale items. The scale had excellent internal consistency and concurrent validity according to its original validation study^22^. TFS was previously used in an Asian sample similar to the present and had demonstrated good concurrent validity^23^. It has a Cronbach alpha of 0.70 and 0.74 at the pre-COVID-19 and lockdown time points, respectively.

For the GDS and GAI, higher scores corresponded to worse outcomes, whereas lower scores in TFS corresponded to worse outcomes. The individual items from these questionnaires were used as nodes in the network analyses.

### Statistical analyses

To provide an overview of the three outcome measures, we assessed the changes in total scores across time using paired-samples t-tests. Then, we constructed partial correlation networks separately at both time points, using the graphical Lasso based on an extended Bayesian information criterion (EBICglasso) option within the R package qgraph^24^. This approach reduces small partial correlations to zero such that they do not appear in the final network; false-positive edges are eliminated in the process, thus leaving only robust edges in the final network. The spearman’s correlation method was used in view of the ordinal data from the TFS items. Although several graph theory metrics can be derived from these networks, not all are meaningful and interpretable in the current context. Hence, we restricted our analyses and interpretations to metrics such as global strength, edge values, nodal strength centrality and bridge centrality, which were more relevant in the current context. Global strength refers to the sum of the absolute values of all edges in the network. The edge value represents the partial correlation between a pair of nodes after controlling for the influence of other nodes. The nodal strength centrality is the sum of these absolute values from all edges that connect to the node. Bridge strength centrality indicates the sum of all absolute edge values from a node that connects to other communities^25^. In the current study, the affective (GDS and GAI items) and social isolation nodes (TFS items) are assigned to two different communities, to test the connectivity between affective symptoms and social isolation.

In order to examine the stability of the network metrics, we carried out a case-dropping bootstrapping procedure with 1000 bootstraps and computed the correlation stability (CS) coefficients for the edge values. The CS coefficient is defined as the maximum proportion of cases that can be dropped while maintaining a 95% probability that the correlation of the network metrics between the sample with and without dropouts is at least 0.70. It was previously suggested that the CS coefficients should be at least 0.25^26^. These procedures were executed using the R package bootnet^26^.

Finally, we carried out paired network comparison tests using the R package NetworkComparisonTest^27^ to compare the edge values, nodal and bridge strength centrality, and global strength between both time points. The statistical significance of these differences across time was determined using a null distribution generated from 5000 iterations of a permutation test. Statistical significance was set at *p* < 0.05. All analyses were carried out in R 4.0.0. There were 24 missing data points out of a total of 34,358 data points. Missing data was handled via pairwise deletion during the network estimation process.

The ‘qgraph’ function within the qgraph R package^24^ was used in the visualization of the constructed networks. To facilitate visual comparison, the nodal positions in all three networks are fixed to a common layout, as determined by averaging layouts of the Pre-COVID-19 and lockdown networks, using the averageLayout function within the qgraph package. The nodes are arranged such that strongly connected nodes would cluster in the middle, whereas less connected nodes are located in the periphery of the network.

## Results

### Changes in total scores across time

**Figure 1:**
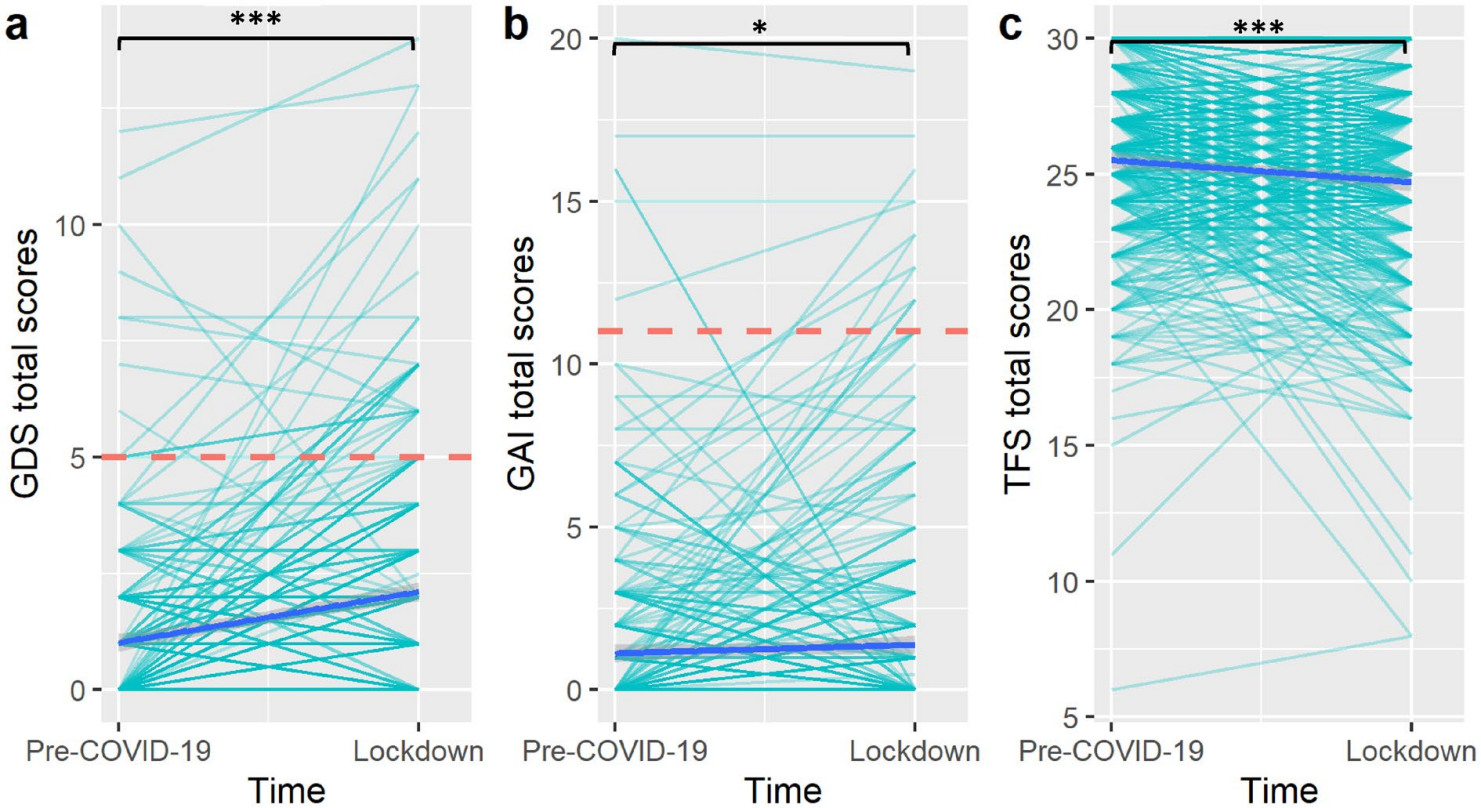
Changes in the total scores of (**a**) geriatric depression scale, (**b**) geriatric anxiety inventory and (**c**) the friendship scale across time. Each light blue line represents the trajectory of a single participant. The dark blue line represents the mean trajectory. The red dotted lines represent the clinical cutoffs for the geriatric depression scale and geriatric anxiety inventory. *p < 0.05, ***p < 0.001.

Table 1 shows the descriptive statistics of the three questionnaires’ total scores at both timepoints. Figure 1 illustrates the changes across time in these total scores. Paired-samples t-tests revealed GDS (t = 10.61, *p* ≤ 0.001, cohen’s d = 0.52) and GAI (t = 2.30, *p* = 0.022, cohen’s d = 0.11) scores had increased significantly, whereas TFS scores had decreased significantly (t = 5.14, *p* ≤ 0.001, cohen's d = 0.25) during the lockdown period.

**Table 1:** Descriptive statistics.

Variable	M	SD
1. GDS_pre-COVID-19_	1.02	1.76
2. GAI_pre-COVID-19_	1.12	2.58
3. TFS_pre-COVID-19_	25.51	3.22
4. GDS_lockdown_	2.11	2.30
5. GAI_lockdown_	1.38	3.14
6. TFS_lockdown_	24.70	3.57

*GDS* geriatric depression scale, *GAI* geriatric anxiety inventory, *TFS* the friendship scale.

### Network analyses

**Figure 2:**
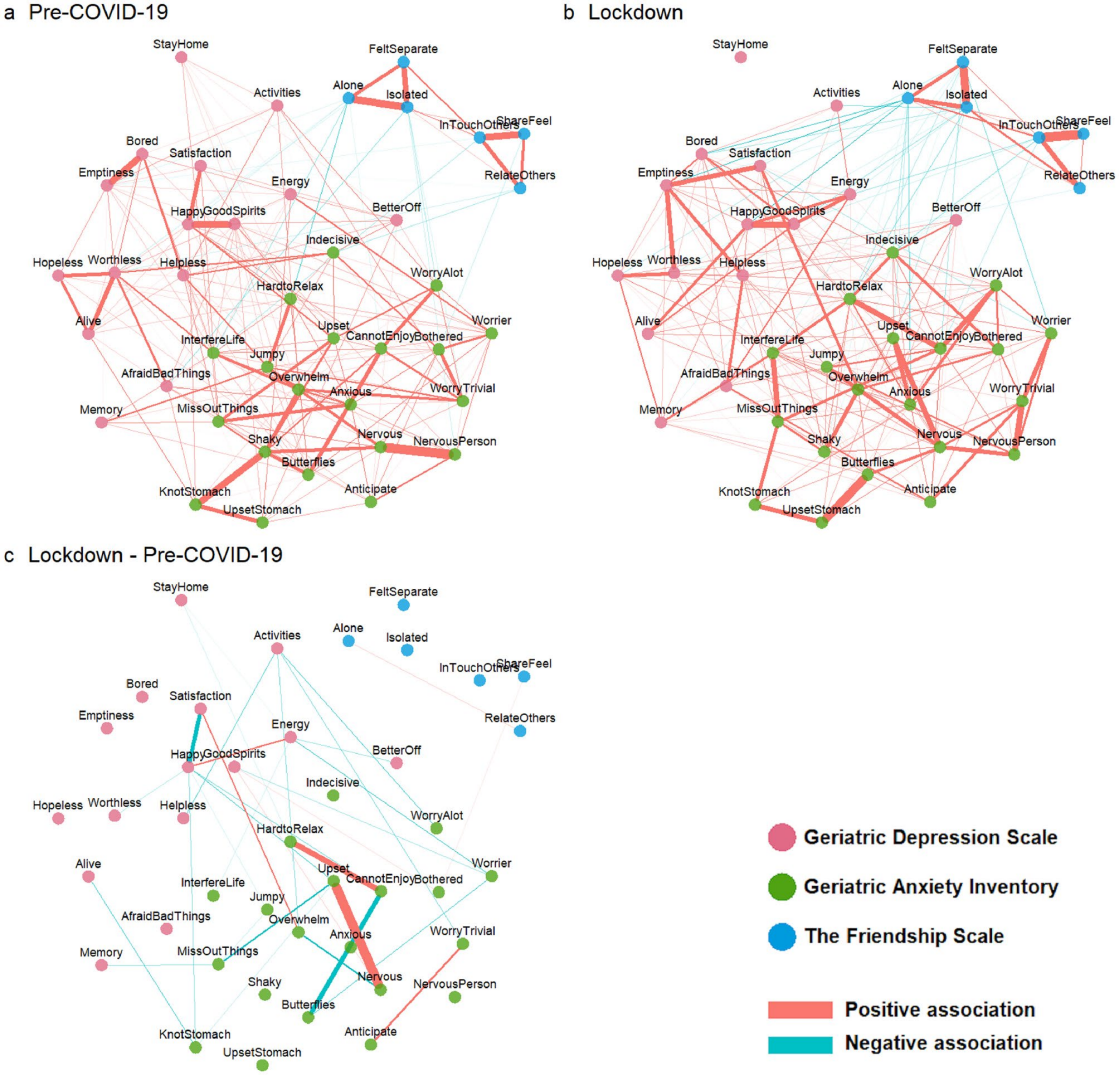
Partial correlation networks depicting items from the geriatric depression scale, geriatric anxiety inventory and the friendship scale at (**a**) Pre-COVID-19 and (**b**) lockdown. Differences across time in edges values are presented in the (**c**) Lockdown—Pre-COVID-19 network. Only the edges with significant changes (uncorrected *p* > 0.05) across time, as determined by the paired network comparison test, are shown. “Positive association” and “Negative association” in the context of (**c**) meant that the edges became more positive or negative, respectively, across time. Thicker lines corresponded to stronger associations.

**Figure 3:**
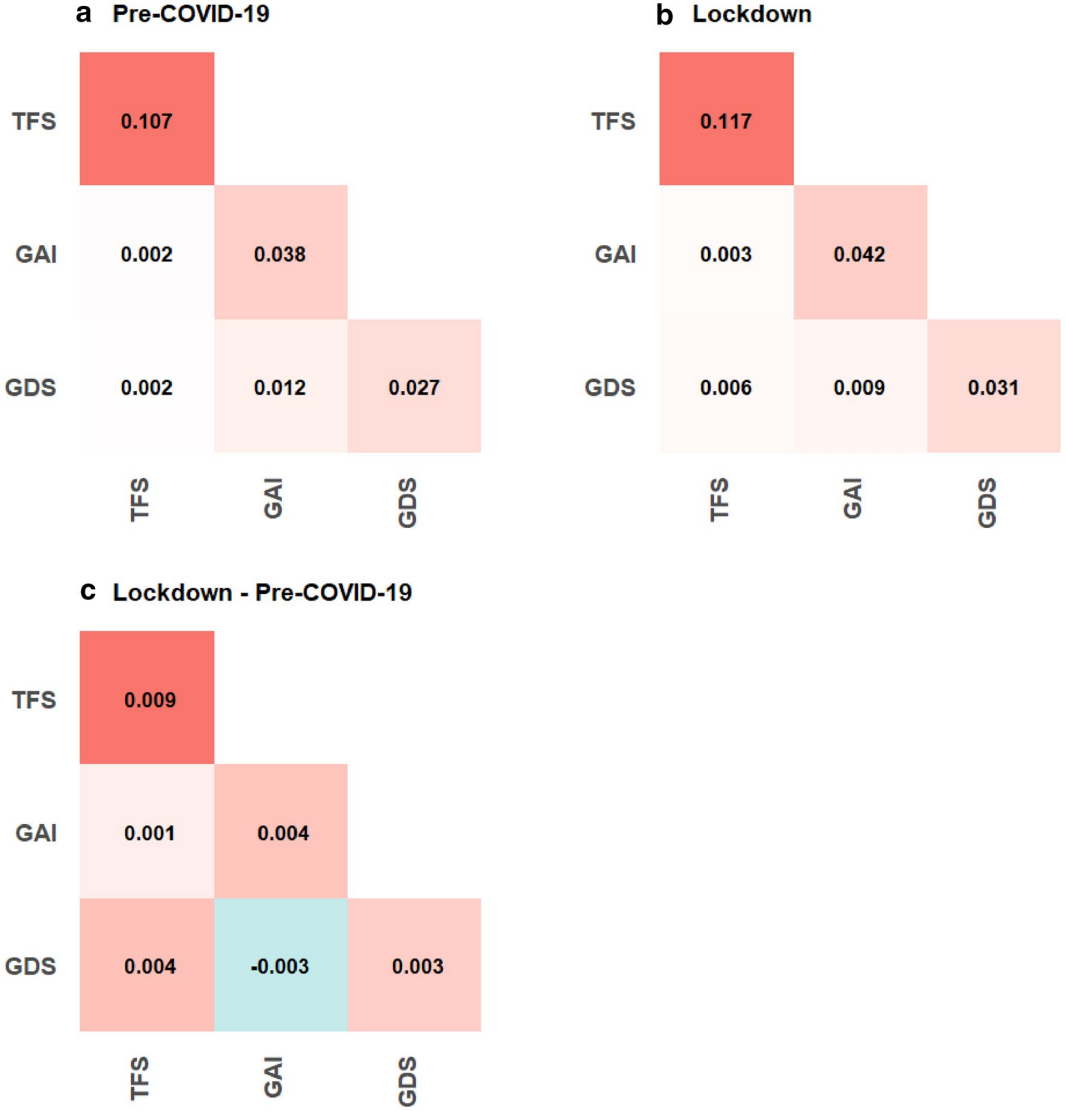
Average (absolute) edge values between and within questionnaires at (**A**) pre-COVID-19, (**B**) Lockdown and (**C**) of the difference between both timepoints. The diagonal represents the average (absolute) edges values within questionnaires.

The CS coefficients for the edges at pre-COVID-19 and lockdown were 0.39 and 0.44, respectively; the CS coefficients for the centrality strength were 0.44 and 0.44 at both time points. These results suggest that the orders of edge and centrality strengths are interpretable with some care^26^. Figures S1 and S2 in the Supplementary Materials further show how the average correlation of the edges and nodes between the original and bootstrapped samples varies with the proportion of sampled cases. Figure 2a and b illustrate the pre-COVID-19 and lockdown networks. These networks consisted of consisted of 228 and 260 non-zero edges, respectively. Figure 3A and B provide a summary of the within- and between-questionnaire nodal connectivity at both timepoints. As shown in the figures, the average absolute edge values between-questionnaires were low (i.e. 0.002 to 0.012) compared to those of within-questionnaires (i.e. 0.027 to 0.117)—suggesting that within-questionnaire nodal connections dominated the network and relatively fewer and weaker connections exist between any two questionnaires. Table 2 shows the 10 strongest edges at each time point. These edges occurred generally between highly similar nodes within the same questionnaire.

**Table 2:** The 10 strongest edges at each time point.

Pre-COVID-19		Lockdown	
Edge	Weight	Edge	Weight
Nervous ↔ nervous person	0.327	Share feel ↔ in touch others	0.330
Isolated ↔ alone	0.276	Butterflies ↔ upset stomach	0.324
Shaky ↔ knot stomach	0.271	Isolated ↔ felt separate	0.296
Good spirits ↔ happy	0.268	Good spirits ↔ happy	0.250
Share feel ↔ in touch others	0.267	Worry trivial ↔ nervous person	0.249
Emptiness ↔ bored	0.265	Nervous ↔ upset	0.243
Isolated ↔ felt separate	0.226	Interfere life ↔ miss out things	0.232
Alive ↔ worthless	0.210	Hard to relax ↔ cannot enjoy	0.224
Cannot enjoy ↔ butterflies	0.199	Satisfaction ↔ emptiness	0.213
Relate others ↔ in touch others	0.196	Relate others ↔ in touch others	0.211

**Figure 4.**
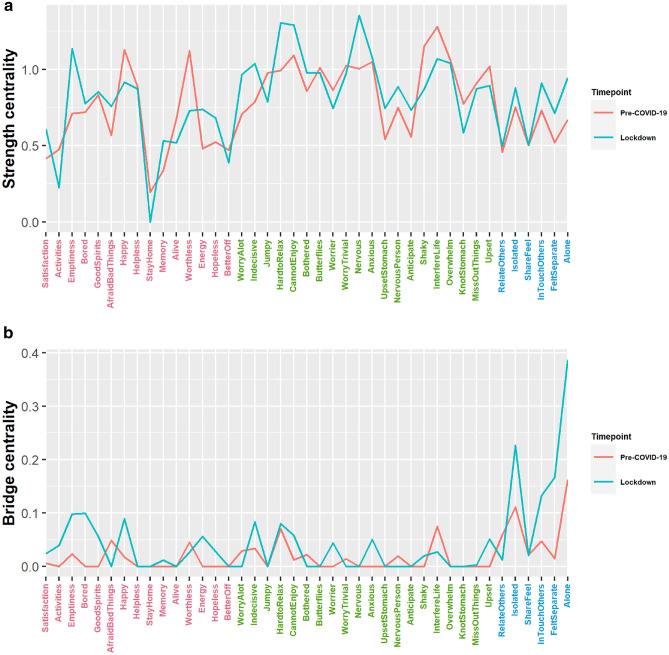
(**A**) Strength centrality and (**B**) bridge centrality of all nodes across both time points. The red, green and blue axis labels correspond to the items from the geriatric depression scale, geriatric anxiety inventory and the friendship scale, respectively.

Moving on to comparisons across time, global strength was not significantly different (*p* = 0.72) at pre-COVID-19 and lockdown. Overall, the average absolute edge values have increased within and between questionnaires, except between GDS and GAI (see Fig. 3C). A closer examination in the differences across time in individual nodes (see Fig. 4) would suggest that various nodes had increased and decreased in nodal and bridge strength centrality across time, though none of these changes emerge statistically significant from the permutation tests. Across both timepoints, the nodes from the GAI tend to be higher in centrality than those of the GDS and TFS. Narrowing down to the individual edges, our permutation analyses identified 34 edges that had changed significantly across time (see Fig. 2c).

### Interpretations

There could be many possible interpretations arising from the results at the level of the individual nodes and edges. For brevity sake, we shall discuss a few. First, the ‘StayHome’ (i.e. Do you prefer to stay at home, rather than going out and doing new things?) and ‘activities’ (i.e., Have you dropped many of your activities and interests?) nodes were located toward the periphery of the network plot, reflecting their low centrality indices. In particular these centrality indices were even lower in the lockdown time point, suggesting that these nodes were highly irrelevant in the assessment of depression at the lockdown time point. This is not surprising since staying home during the lockdown was no longer a personal choice that was influenced by one’s mood, but a mandatory lockdown requirement. Likewise, the dropping of many activities and interests should be seen as the direct consequence of the lockdown restrictions, and was unlikely to be attributed to mood changes. Indeed, we observed that a few edges that connected between the ‘activities’ node and depressive/anxiety nodes had significantly weakened in the lockdown time point (see Fig. 2c). Next, the edge between ‘Upset’ (i.e., I often feel upset) and ‘Nervous’ (i.e., I often feel nervous) registered the largest change across time in the network—suggesting that feelings of nervousness and upset have become more intimately linked during the lockdown. Speculatively, the experience of nervousness could have been appraised more negatively, possibly due its increased likelihood to accompany other socio-affective consequences, as reflected by the increased centrality of ‘Nervous’ across time.

Moving on to the results at the questionnaire level. The TFS nodes appeared to be weakly associated with the GDS and GAI nodes at pre-COVID-19, as indicated visually by the smaller number and magnitude of edges (see Fig. 2a), and also objectively via the relatively low average absolute edge values (see Fig. 3A). However, these TFS nodes became more visibly connected to the GDS and GAI nodes at lockdown (see Fig. 2b); these connections were also associated with higher average absolute edge values (see Fig. 3B) and bridge strength centrality (see Fig. 4B). Taken together, this suggests that social isolation had a greater influence on affective symptoms at lockdown than at pre-COVID-19. While connectivity has increased within all three questionnaires at lockdown, as indicated by the increased average edge values, some decoupling between the GDS and GAI nodes has also occurred. For instance, multiple edge connections between GDS and GAI nodes have weakened significantly (see Fig. 2c) and the average edge values between GDS and GAI have also decreased across time (see Fig. 3C).

## Discussion

The current study investigated how a COVID-19 lockdown has altered the dynamics between depression, anxiety, and social isolation. Overall, participants' well-being had worsened significantly in these socio-affective domains during the lockdown. The lockdown has strengthened the association between social isolation and affective symptoms. Importantly, our results also indicated that depression, anxiety, and social isolation nodes were more strongly coupled within their respective domains during the lockdown, despite the slight decoupling between depression and anxiety. This strong coupling meant that across individuals, the appearance of any single affective symptom is likely to coincide with the appearance of other related symptoms. Likewise, the appearance of any single social isolation-related perception is also likely to coincide with those of other related perceptions. Our network-based findings provide novel and useful perspectives on the increased vulnerability to psychopathology during this period.

While it is expected that perceived social isolation would have increased during this period of time, it is somewhat alarming that such perceptions had become more strongly associated with affective symptoms. There are two likely explanations for this. First, the same level of perceived isolation may have become more distressful or appraised more negatively during the lockdown. For instance, our results showed that the thought of being ‘alone’ has become more likely to co-occur with several depression nodes, including feelings of ‘emptiness’. Speculatively, participants were more likely during the lockdown to overinterpret or overgeneralize their feelings of loneliness as emptiness in their life. Second, the lockdown might have weakened protective factors that could have mitigated affective symptoms. Hence, the same level of perceived social isolation has become more damaging in the absence of these protective factors. For instance, physical activity, which has been drastically limited during the lockdown, was known to protect older adults from depression^28^. In particular, previous research has shown that even a single session of physical exercise, can significantly enhance the emotional regulation^29^ that is needed to buffer against affective dysfunction. Social support is another such protective factor that has weakened; in fact, it might have a double role in the current context. While the difficulty of accessing social support during the lockdown has increased the perceptions of social isolation, the lack of social support also made it difficult for one to cope with stress^30^ and regulate emotions^31^. In general, these weakened protective factors could amplify the effects of most if not all etiological factors associated with affective disorders, beyond that of social isolation.

Across individuals, the lockdown has increased the coupling of symptoms within the depression or anxiety syndrome. This suggests that affective symptoms are more likely to appear concurrently with other symptoms as part of a larger cluster of depression or anxiety symptoms, alluding to a steeper developmental trajectory of affective dysfunction. The weakened protective factors, as discussed above, especially in relation to compromised emotional regulation, could have allowed internalizing symptoms, such as those associated with depression and anxiety to escalate more rapidly^32,33^. This increased coupling of within-syndrome symptoms could also be attributed to a ‘third variable problem’; that is, the tight coupling between these symptoms was merely the consequence of them being triggered by the same extraneous variable. In the current context, this ‘third variable’ could manifest as the fear, uncertainty, and daily stressors associated with the COVID-19 pandemic. Regardless of the cause, the increased within-syndrome coupling among affective symptoms reflects an increased vulnerability to affective dysfunction. Interestingly, despite the increased within-syndrome coupling, the syndromes appeared to have decouple from each other. Speculatively, this could mean that depression and anxiety are triggered by different lockdown-related factors. For instance, the fear of getting infected with COVID-19 may be more specific in triggering anxiety symptom^34^, whereas the lockdown-related isolation may aggravate depression symptoms^35^ more so than those of anxiety.

Our findings present important implications in the wider public health context. First, we provided valuable longitudinal evidence pointing to the deteriorating mental health situation among community-dwelling older adults. Second, in view of the fact that the COVID-19 pandemic has overstretched healthcare resources, it has become even more important to identify efficient intervention targets, to maximize treatment gains with minimal resources. To this end, we have identified several symptoms that were highly central in the network such as nervousness, anhedonia, perceptions of loneliness and emptiness. Given their centrality in the network, the successful treatment of these symptoms would likely ‘switch off’ other connected symptoms^13^, thus accelerating the recovery process. Nevertheless, efficacy of such a targeted treatment approach needs to be empirically tested as previous findings in this area have been mixed. While two intervention studies have shown that the centrality of a treated symptom predicts overall improvement in the network of symptoms associated with social anxiety disorder^36^ and posttraumatic stress disorder (PTSD)^37^, a more recent study^38^ utilizing another PTSD sample did not observe the centrality of the treated symptom to be significantly associated with improvements in the other symptoms.

Next, our network maps identified certain low centrality symptoms such as ‘StayHome’ and ‘Activities’. While these ‘symptoms’ have been shown to contribute significantly in classifying depressed and healthy controls in the validation studies that occurred prior to COVID-19, in light of the ongoing COVID-19 restrictions, these ‘symptoms’ may no longer reflect a mood disturbance. This issue extends beyond the GDS—for instance the Hamilton Depression Rating Scale has an item assessing daily activities^39^. Thus, epidemiological surveys that used such scales to assess depression during the COVID-19 lockdown periods may have inadvertently over-estimated the severity or prevalence of depression symptoms.

The current findings are subjected to some limitations. First, given that the COVID-19 situation and its associated lockdown can vary very differently across countries and cities, it would be difficult to generalize our results to other older adult populations in other countries and cities. Second, given that the data collection for the pre-COVID-19 time point spanned across almost two years, the follow-up durations are highly variable across participants and may potentially confound our results. Subjects with longer follow-up durations are more likely to experience significant social and/or personal events that would influence their levels of depression, anxiety, and social isolation between the pre-COVID-19 baseline and the lockdown timepoints. This meant that the changes in these socio-affective variables between both timepoints are less likely to be attributed to the COVID-19 lockdown for these subjects. Third, although the CS coefficients were satisfactory (≥ 0.25), they were still below the recommended 0.50^26^. This meant that the associations between nodes across the study participants were not as stable as desired—it is possible that a small group of participants with extreme scores are driving these associations. Thus, the findings of the current study would require validation in larger sampled studies. Finally, while it is tempting to interpret these results from a within-person perspective (i.e., symptom becomes more tightly coupled within a person), this would not be justified because our results are derived from between-subject networks. Nevertheless, these between-subject findings do reveal on a macroscopic level, the general structural changes of affective symptomology in relation to perceived social isolation during a lockdown. These networks also generate testable hypotheses on the psychiatric developmental trajectories shared by individuals^11^.

## Supplementary Information

Supplementary Information.

## Data Availability

The datasets generated during and/or analysed during the current study are not publicly available due to the conditions of our ethics approval but are available from the corresponding author on reasonable request.

## Code availability

The R code for executing these analyses and generating the figures are available at https://osf.io/gu4wd/files/.
