# Supplementary material for: COVID-19 lockdown has altered the dynamics between affective symptoms and social isolation among older adults: results from a longitudinal network analysis
Source: Sci Rep. 2021 Jul 19;11:14739. doi: 10.1038/s41598-021-94301-6 (PMC8289844; doi:10.1038/s41598-021-94301-6)
Supplement: Supplementary file 1 — Supplementary Information. [file 41598_2021_94301_MOESM1_ESM.docx]

a)


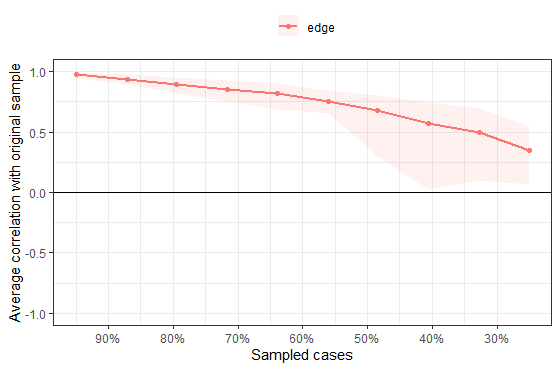


b)


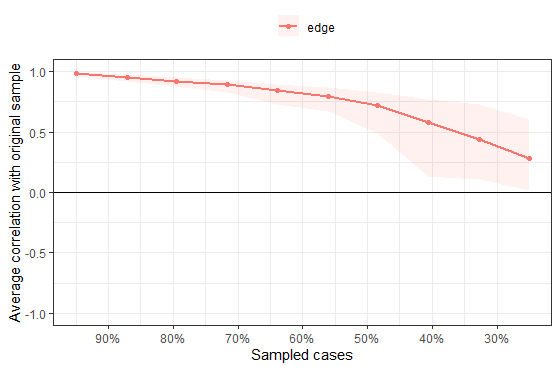


Figure s1. plots showing the correlation stability of edges at the a) pre-COVID-19 and b) lockdown time points. The dark red line represents the average correlation coefficient enclosed with faint red-ribbon representing the 95% confidence interval

a)


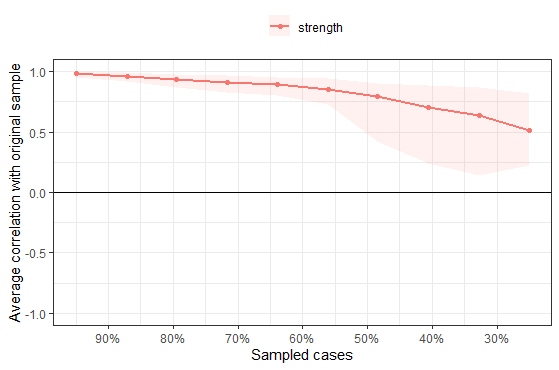


b)


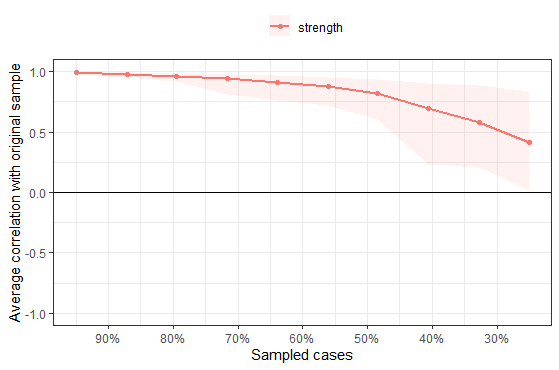


Figure s2. plots showing the correlation stability of centrality strengths at the a) pre-COVID-19 and b) lockdown time points. The dark red line represents the average correlation coefficient enclosed with faint red-ribbon representing the 95% confidence interval
